# Environmental Noise Classification with Inception-Dense Blocks for Hearing Aids

**DOI:** 10.3390/s21165406

**Published:** 2021-08-10

**Authors:** Po-Jung Ting, Shanq-Jang Ruan, Lieber Po-Hung Li

**Affiliations:** 1 Department of Electronic and Computer Engineering, National Taiwan University of Science and Technology, Taipei 106, Taiwan; m10802108@mail.ntust.edu.tw (P.-J.T.); sjruan@mail.ntust.edu.tw (S.-J.R.); 2 Department of Otolaryngology, Cheng Hsin General Hospital, Taipei 112, Taiwan; 3 Faculty of Medicine, and Institute of Brain Science, National Yang-Ming Chiao-Tung University, Taipei 112, Taiwan; 4 Department of Medical Research, China Medical University Hospital, China Medical University, Taichung 404, Taiwan; 5 Department of Speech Language Pathology and Audiology, College of Health Technology, National Taipei University of Nursing and Health Sciences, Taipei 112, Taiwan

**Keywords:** hearing aids, environmental noise classification, deep learning, convolutional neural networks

## Abstract

Hearing aids are increasingly essential for people with hearing loss. For this purpose, environmental noise estimation and classification are some of the required technologies. However, some noise classifiers utilize multiple audio features, which cause intense computation. In addition, such noise classifiers employ inputs of different time lengths, which may affect classification performance. Thus, this paper proposes a model architecture for noise classification, and performs experiments with three different audio segment time lengths. The proposed model attains fewer floating-point operations and parameters by utilizing the log-scaled mel-spectrogram as an input feature. The proposed models are evaluated with classification accuracy, computational complexity, trainable parameters, and inference time on the UrbanSound8k dataset and HANS dataset. The experimental results showed that the proposed model outperforms other models on two datasets. Furthermore, compared with other models, the proposed model reduces model complexity and inference time while maintaining classification accuracy. As a result, the proposed noise classification for hearing aids offers less computational complexity without compromising performance.

## 1. Introduction

The number of people who have hearing loss has been increasing. Nevertheless, the number hearing aid users is not obviously increasing at the same rate [1]. According to the latest statistics from the World Health Organization (WHO), around 466 million people worldwide suffer from hearing loss, and only 17% use hearing aids [2]. Wearing hearing aids compensates for the hearing loss of hearing-impaired listeners, providing many benefits for them [3]. However, one of the common complaints of hearing aid users is the hearing aid’s inability to reduce ambient noise completely; instead, they cause ambient noise to be amplified along with the human voice [4]. The hearing aid’s inability to reduce ambient noise completely is due to their operation in various, irregular, and random environmental sound scenarios [5]. Speech enhancement algorithms are an essential technique for hearing aids. At present, deep neural network-based speech-enhancement methods have been widely adopted and have shown significant performance advantages over conventional speech enhancement techniques in complex noise environments. Several studies [6,7,8] have noted that using machine-learning-based environmental noise classification techniques to classify environmental noise, before using speech enhancement, can improve speech enhancement algorithm performance. Thus, the environmental noise classification (ENC) algorithms for hearing aids deserve attention.

**Figure 1:**
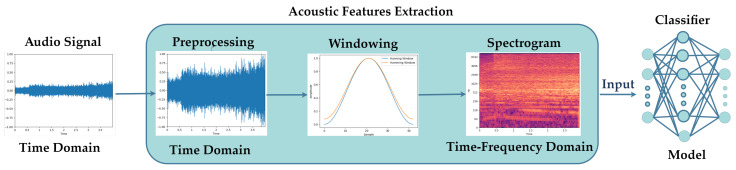
Typical processing flow of an environmental sound classification system.

Figure 1 shows the typical processing flow of environmental sound classification (ESC), consisting of two essential components: an acoustic feature extractor and a classifier. In order to extract useful acoustic features, audio signals are first pre-processed in the time domain, including normalization and silence reduction. Then, the audio signals are converted to the audio frames with a cosine window function (Hanning or Hamming window). Finally, features are extracted from each audio frame, and the extracted acoustic features are fed into the classifier for training and testing. The result of classification is the prediction probability for which audio signals belong to which type of sound. In previous works [9,10], traditional acoustic feature techniques, such as mel frequency cepstral coefficients (MFCC), linear predictive coding (LPC), and perceptual linear predictive coefficients (PLP) are already used in the ESC field. However, using more discriminative representations, such as mel filter bank features [11], wavelet-based features [12], and Gammatone features [13], can achieve better performance. Many typical machine learning algorithms have been used to classify sounds, such as support vector machine (SVM) [14], Gaussian mixture model (GMM) [15], and k-nearest neighbors (KNN) [16] for ESC tasks. However, the performance of these typical machine learning algorithms is still unsatisfactory. One reason is that typical classifiers cannot capture time and frequency features when applied to spectrogram-like input.

In recent years, deep neural networks (DNNs) have succeeded in ESC tasks and have provided better performance than typical machine learning algorithms [17,18]. For audio signals, DNNs can extract features from raw data or simple hand-crafted features. However, the deep fully-connected architecture of DNNs is not robust for transformative features. Several studies have shown that convolution neural networks (CNNs) can capture relevant features from an image. Furthermore, training CNN models with spectrogram-like features from environmental sounds demonstrates significant performance benefits over other methods [16,19,20]. However, existing high-accuracy CNNs also have a higher model complexity, leading to more computational complexity, which means more floating-point operations (FLOPs). A higher number of FLOPs is directly proportional to CPU consumption [21].

Although much work has been done on environmental sound classification, environmental noise classification for hearing aids is still critically lacking. The computational resources of hearing aids pose the major limitation that they cannot compute a large set of high-level features, and such features cannot be fed into a sophisticated environmental sound classifier [7]. One way to overcome these limited computational resources is to train the model on the server; the trained model can then be downloaded to hearing aids, which can then perform the environmental sound classification in real-time. However, the CNN model used in the ESC tasks mentioned above have a high computational complexity. Hence, reducing the computational model complexity while maintaining the model accuracy is essential.

This study attempts to provide a new model architecture for environmental noise classification on hearing aids that minimizes complexity by utilizing Inception-Dense block and depthwise-separable convolution. In order to verify the effectiveness of the proposed model, two index data analyses are performed: 1. FLOP counts are taken, representing the model’s computational complexity; 2. inference time—the running time for the model on a smartphone—is measured. For this objective to be achieved, the paper is structured as follows. Section 2 is a review of the literature, including the current research on feature extraction, environmental sound classification, and environmental noise classification for hearing aid. Section 3 provides a detailed introduction of proposed methods. In Section 4, the setting of the experiments on the UrbanSound8K dataset and the hearing aids noisy sound (HANS) dataset are described. Section 5 compares the performance of the proposed approach with previous models used in environmental noise classification and environmental sound classification. The conclusions and perspectives of future work are presented in the last section.

## 2. Related Work

This section first introduces the feature which will be used as the input of the environmental noise classifier. Then, the current research on environmental sound classification and environmental noise classification will be introduced.

### 2.1. Time-Frequency Representations for Noise Signal

Several types of desirable information are contained in raw audio data, such as the short-time Fourier transform (STFT) spectrogram, log-scaled mel-spectrogram, and gammatone spectrogram. This paper uses the log-scaled mel-spectrogram as the input of our proposed model. The reasoning for choosing this feature is stated below.

With a growing amount of evidence, the log-scaled mel-spectrogram outperforms other features in environmental sound classification tasks. Huzaifah [22] compared five signal processing methods, such as STFT-spectrogram, log-scaled mel-spectrogram, constant-Q transform (CQT), continuous wavelet transform (CWT), mel frequency cepstral coefficients (MFCCs), using these features as the input of two different models. The results indicate that the model that consisted of three convolution layers and two fully-connected layers, and which used the log-scaled mel-spectrogram as a feature, performed the best. Su et al. [23] resulted in the finding that the performance of the log-scaled mel-spectrogram is better than the gammatone spectrogram and the mel-spectrogram. Furthermore, and more essential, the log-scaled mel-spectrogram is computationally more efficient for real-time implementation than the CQT spectrogram, CWT scalogram, and MFCC.

The log-scaled mel-spectrogram generation process includes:

1. Signal pre-processing.
2. A Fourier transformation to obtain the signal spectrogram.
3. Mapping of the spectrogram into a mel-spectrogram through triangular overlapping windows whose center frequencies are distributed on the mel scale. The function *B* for computing the *m*th mel-frequency from frequency *f* in Hertz and its inverse $B^{-1}$ are given by [24]:
  (1) $$B\left(f\right)=2595\log_{10}\left(1+\frac{f}{700}\right),$$
  (2) $$B^{-1}\left(m\right)=700\left(10^{\frac{m}{2595}}-1\right).$$
4. Taking a log calculation (decibles) on the mel spectrogram,
  (3) $$LM\left(n\right)=\log(\sum_{k=0}^{K}H_{n}\left(k\right)*{\left|F(k)\right|}^{2}),\;n=1\ldots N,$$
  where $H_{n}\left(k\right)$ denotes the amplitude of the *n*th filter at frequency bin *k*, ${\left|F\left(k\right)\right|}^{2}$ denotes the FFT power spectrum.

### 2.2. Conventional Noise Classification Algorithms

Many conventional classification algorithms are used in noise classification. The KNN classification algorithm calculates the distance of the new input data from the *k* nearest points which determines the class of a new input data point. It is suitable for simple classification problems with basic training features. As the number of training features increases, KNNs computational complexity and time increases. SVM and Neural Networks [25] are feasible when there is a clear margin of separation between classes and are more effective in high dimensional spaces. However, SVM performs poorly when the data set has more noise, or the data set is too large. HMM is a widely used statistical method for speech recognition. One major advantage of HMMs over the previously described classifiers is that they account for the temporal statistics of different states’ occurrence in the features.

Nordqvist and Leijon [26] used the HMM and vector quantizer to classify three kinds of auditory environments (traffic noise environment, pure voice environment, and babble noise environment). Büchler et al. [27] selected characteristic parameters from the perspective of acoustic scene analysis to distinguish four different acoustic scenes (voice, noisy voice, noise, and music). Those characteristics have been evaluated together with different pattern classifiers. Simple classifiers, such as rule-based and minimum-distance classifiers, have been compared with more complex approaches, such as Bayes classifier, neural network, and HMM. Abe et al. [28] selected eleven features and used Bayes classifier, SVM, and Logistic regression to classify four kinds of auditory environments (speech, speech in noise, noise, and classical music). The conventional noise classification algorithms for hearing aids focused on voices and music. There is a paucity of conventional noise classification algorithms for hearing aids on the sound field outside of voices and music.

### 2.3. Deep Convolutional Neural Network

A CNN is a deep learning technology based on supervised learning and is widely used for image processing while maintaining the spatial information of the image. Recently, several deep learning methods for environmental sound classification have been conceived. Piczak [16] created a two-channel feature by applying the log-scaled mel-spectrogram and its delta information as the inputs of his CNN model, and the model achieved 73% accuracy on the UrbanSound8K dataset. Salamon and Bello [19] compared different data augmentations that could influence the accuracy of each class. They used the log-scaled mel-spectrogram as the input of the CNN model, and the accuracy was 79%. Zhang et al. [29] applied mixup and data augmentation to ESC tasks. They used the log-scaled mel-spectrograms and their delta information a as two-channel feature, and a similar CNN architecture to VGG net; the accuracy achieved was 82%. Palanisamy et al. [20] computed three different window sizes and hop lengths as three-channel features for the input to Inception [30], ResNet [31], and DenseNet [32]. The result showed that DenseNet is the best of the three.

Although there is much discussion on environmental sound classification based on CNNs in the literature, there is a paucity of noise classification algorithms for hearing aids based on CNNs. Singh and Joshi [33] used log-scaled mel-spectrograms as the input of a similar VGG net to classify background sound in a speech audio segment. Park and Lee [34] processed the spectrogram image through a sharpening mask and median filter, which was then used as the input of the CNN. However, the dataset is not public, and the model structure is not clear. Roedily et al. [35] used MFCC as the input of a CNN-LSTM model and an inference model on a smartphone. In order to evaluate the performance of our proposed model, the model is evaluated against the environmental sound classifications of other CNNs on the UrbanSound8k dataset, and is then evaluated against the noise classification of the Roedily and Singh models on the HANS dataset.

## 3. Proposed Methodology

### 3.1. Inception Block with Dense Connectivity

**Figure 2:**
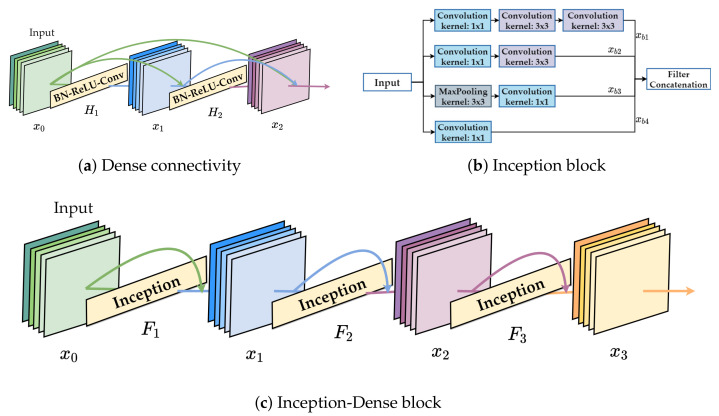
(**a**) Dense connectivity. (**b**) Inception block. (**c**) Inception-Dense block. (**a**,**b**) have two common points: the first is that they all use 1x1 convolution as a BottleNeck layer; the second is that they all use filter concatenation connections. The difference between (**a**,**b**) is that the input tensor directly passes to the output tensor without any other middle operations in (**a**).

Figure 2 shows three blocks; the dense connectivity [32], Inception [30], and Inception-Dense blocks. The advantage of dense connectivity is that it bypasses connections. It can reuse feature maps from the previous layers. Figure 2a illustrates the layout of the dense connectivity block. The $\ell_{th}$ layer receives the feature-maps of all preceding layers, and $x_{0},\ldots ,x_{\ell -1}$ are used as input to $H_{\ell }\left(.\right)$:

(4) $$x_{\ell }=H_{\ell }\left(\left[x_{0},x_{1},\ldots ,x_{\ell -1}\right]\right),$$

where $\left[x_{0},x_{1},\ldots ,x_{\ell -1}\right]$ refers to the concatenation of the feature-maps produced in layers 0,…,ℓ−1. $H_{\ell }\left(.\right)$ is a composite function of three consecutive operations: batch normalization (BN), followed by a rectified linear unit (ReLU) and a 3×3 convolution (Conv). However, various frequency bands and time intervals are important characteristics of the individual sound types. The advantage of Inception is that a combination of different kernel sizes can take multi-level feature maps. Using multi-level feature maps from multiple filters improves the performance of the network. Moreover, all the architectures prior to Inception performed convolution on the spatial and channel-wise domains. Figure 2b illustrates the layout of the Inception block. By performing the 1×1 convolution, the Inception block performs cross-channel correlations, ignoring spatial dimensions, followed by cross-spatial and cross-channel correlations via the 3×3 and 5×5 filters.

Therefore, dense connectivity is utilized to connect all Inception blocks on the proposed structure. As shown in Figure 2c, Inception blocks are connected by dense connectivity, allowing each block to receive input directly from its previous block. Similar to DenseNet, a layer in the proposed model implements a non-linear transformation $F_{\ell }\left(.\right)$, where *ℓ* is the index of the layer. $F_{\ell }\left(.\right)$ is a composite operation function such as BN, ReLU, Conv, or Pool. The output of $\ell_{th}$ layer is denoted as $x_{\ell }$, which can be defined as :

(5) $$x_{\ell }=concate\left(x_{\ell -1},F_{\ell }\left(\left[x_{b1},x_{b2},x_{b3},x_{b4}\right]\right)\right),$$

where $x_{\ell -1}$ is the output of ℓ−1 layer, $x_{b1},x_{b2},x_{b3},x_{b4}$ are the outputs of each branch in Inception block. $\left[x_{b1},x_{b2},x_{b3},x_{b4}\right]$ is a filter concatenation. It concatenates feature maps along the channel dimension.

### 3.2. Depthwise-Separable Convolution

**Figure 3:**
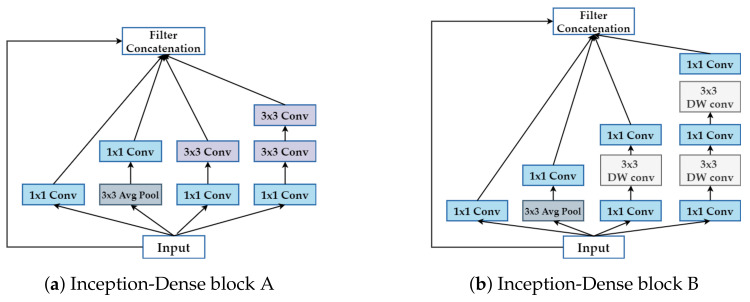
Two kinds of basic Inception-Dense blocks in our model. The differences between the blocks (**a**,**b**): block (**a**) is the traditional Inception block, while block (**b**) replaces the 3 × 3 convolution layer with the 3 × 3 depthwise-separable convolution layer.

Inspired by [36], the standard convolution layer is replaced with a depthwise-separable convolution layer on the Inception-Dense block. The depthwise-separable convolution layer can reduce the total number of operations. It combines a depthwise convolution layer and a 1×1 convolution called a pointwise convolution. The computational cost of a standard convolutional layer, a depthwise convolutional layer, and a pointwise convolutional layer is defined as (6)–(8), respectively. The depthwise-separable convolution layer could be simplified as (9), which is the sum of (7) and (8).

(6) $$W_{in}*H_{in}*C_{in}*K*K*C_{out},$$

(7) $$W_{in}*H_{in}*C_{in}*K*K,$$

(8) $$W_{in}*H_{in}*C_{in}*C_{out},$$

(9) $$W_{in}*H_{in}*C_{in}*\left(C_{out}+K*K\right),$$

where the computational cost depends multiplicatively on the number of input channels $C_{in}$, the number of output channels $C_{out}$, the kernel size K×K, and the size of input feature map $W_{in}\times H_{in}$. The depthwise-separable convolution layer can save 80 to 90 percent of the computation on the 3×3 convolution layer; on the other hand, the accuracy of the model is likely to decrease. Consequently, two types of Inception block with dense connectivity are used on the proposed model, as shown in Figure 3.

### 3.3. Network Structure

**Figure 4:**
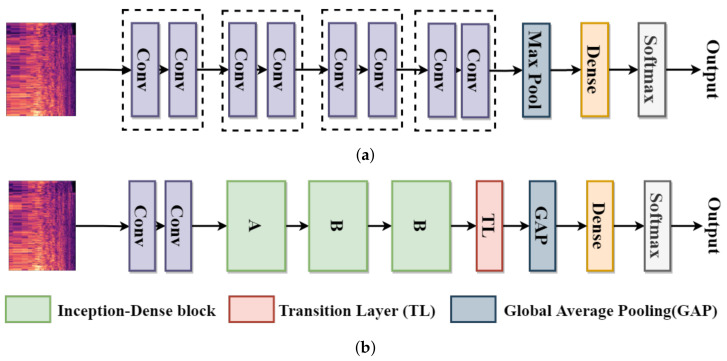
The network structures. (**a**) The state-of-the-art network structure based on VGG Net [33]. (**b**) The proposed network structure.

This section describes the proposed model in detail and shows that the model is compact. The proposed model architecture is comprised of two convolutional layers, three Inception-Dense blocks, and one fully-connected layer. The compared state-of-the-art noise classification network structure is based on VGG Net; the classification accuracy of this network is better than that of others [33]. The network structure is presented in Figure 4a. It stacks two convolutional layers as a module and stacks three or four modules into their network structure. In our model, we also use two initial convolutional layers. Then, the module of the convolutional layer is replaced by the Inception-Dense block; the Inception-Dense block is stacked three times in our network structure. However, considering DenseNet, we use a transition layer after three Inception-Dense blocks. Finally, the fully-connected layer is applied. The proposed network structure is presented in Figure 4b.

**Figure 5:**
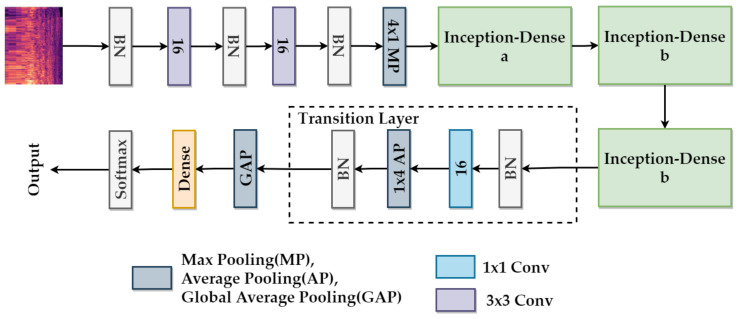
The overall framework of the proposed model. The purple block represents a 3×3 convolution, and the blue block represents a 1×1 convolution. The grey block represents a max-pooling layer, an average pooling layer, or a global average pooling layer. Here only one type of layer on a grey block is present.

The detailed framework of the proposed model is shown in Figure 5. Batch Normalization (BN) is used to normalize the input data at first. Next, two 3×3 convolutional layers are used as a basic feature extractor, each convolutional layer followed by a BN and a Rectified Linear Unit (ReLU) activation function. After the second convolutional layer, a max-pooling layer is used to retain the most prominent features of the feature maps. The pool size of the max-pooling layer is 4×1. Then, three Inception-Dense blocks are used to extract multiple features. The first block uses the Inception-Dense block A. Because of the channel size of the feature maps, all of the 1×1 convolutional layers use 16 filters, and all of the 3×3 convolutional layers use 32 filters in the first block. The second block and the third block use the Inception-Dense block B and the same settings. A BN and a ReLU activation function are followed by a convolutional layer on all Inception-Dense blocks. Next, the transition layer of DenseNet is considered, using a 1×1 convolutional layer and an average pooling layer, followed by a BN and a ReLU activation function.

Inspired by [37], instead of adding a fully-connected layer, a global average pooling layer is added at last, and the vector is fed directly into the softmax layer. The advantage of global average pooling over the fully-connected layer is that it is more native to the convolution structure by enforcing correspondences between feature maps and categories. Thus, the feature maps can be easily interpreted as categories confidence maps. The softmax function is used at the final layer to obtain class probabilities, and the chosen loss function is the cross-entropy loss function. The cross-entropy loss and softmax are used together because they provide a smooth and straightforward gradient, making computations much easier.

The configuration of the proposed model is described in Table 1. As an illustration, the 128×128×1 features are used as the input of the model at the configuration. The model is designed with full consideration of computational efficiency and practicality. The trained model has tiny size, only 1.6 MB. It can be applied to individual devices, including even those with limited computational resources.

## 4. Experiments

### 4.1. Dataset

The proposed model is evaluated on the following datasets: the UrbanSound8K [38] and the Hearing Aids Noisy Sound (HANS) dataset.

**UrbanSound8K**. The UrbanSound8k dataset contains 8732 clips (each of length less than or equal to 4 s). The original sampling rate of each audio clip varies from 16,000 Hz to 44,100 Hz. All audio clips are resampled to a sampling rate of 16,000 Hz, which is the appropriate frequency for signal processing for hearing aids. The dataset is officially split into ten folds and is divided into ten classes: air conditioner (ac), car horn (cr), children playing (ch), dog bark (db), engine idling (ei), gunshot (gs), jackhammer (jh), siren (si), and street music (sm). Note that the dataset is not rearranged.

**Figure 6:**
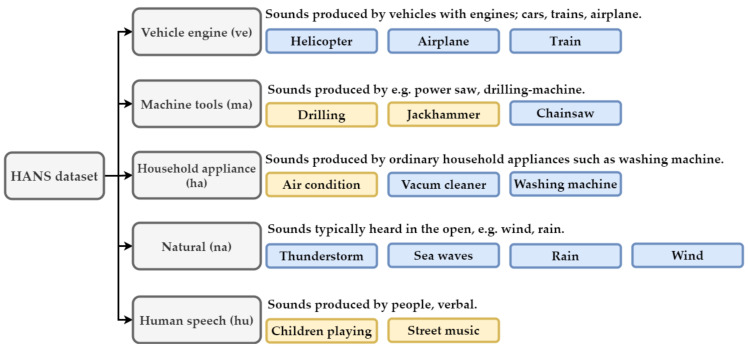
For the hearing aids application, 15 subclasses are grouped into five major categories, and each category is described in detail. A yellow block indicates that the subclass is selected from the UrbanSound8k dataset. A blue block represents that the subclass is selected from the ESC50 dataset.

**Hearing Aids Noisy Sound (HANS)**. Many datasets are available publicly for environmental sounds, but there is no dedicated public dataset with a specific focus on hearing aid applications. Inspired by [5], we built a dataset called the Hearing Aid Noisy Sound (HANS) dataset; it contains sounds considered common and difficult for hearing-aids users. The top five categories of annoying sounds for hearing aids users are verbal human sounds, vehicle sounds, machine tools sounds, natural sounds, and household appliance sounds. The dataset considers the categories of annoying sounds in its description. It selects 5 classes and 10 classes from the UrbanSound8K dataset and the ESC50 [16] dataset, respectively. The 15 classes are separated into five major categories on the HANS dataset as shown in Figure 6. The UrbanSound8K and ESC50 datasets involve nonoverlapping, short clips of environmental sounds. The ESC50 dataset is a weak label dataset, and the length of each audio clip is equal to 5 s. The majority of the audio clip is empty; only a minor part with one sound is included. The Urbansound8k dataset is a strong label dataset. Its sounds are continuous, seldom empty and may contain multiple sounds which are not labeled, such as wind and people speaking. Some sound files on the UrbanSound8K dataset were removed because they conflict with the classes of the HANS dataset. Note that the class of human speech is the only one that contains sound files with speech. If a file in the class of street music only had music, or a file in the class of children playing included the sound of laughter, then these files were removed from the HANS dataset.

Because the UrbanSound8K and ESC50 datasets are small datasets, the official literature uses k-fold cross-validation to evaluate model performance. For that reason, the HANS dataset also uses k-fold validation to evaluate model performance. However, the UrbanSound8K dataset uses 10-fold cross-validation, and the ESC50 dataset uses 5-fold cross-validation. In order to reasonably combine two datasets, the HANS dataset uses 5-fold cross-validation. For using 5-fold cross-validation, two folds of the UrbanSound8K dataset are combined into one fold, such as combining the first fold and the second fold into the first fold of the HANS dataset and combining the third fold and the fourth fold into the second fold of the HANS dataset. The ESC50 dataset directly transferred the original fold to the new fold of the HANS dataset, i.e., by putting the first fold of the ESC50 dataset into the first fold of the HANS dataset. To maintain the appropriate frequency for signal processing on hearing aids, all audio clips are subsequently down-sampled to 16,000 Hz in the HANS dataset.

### 4.2. Data Preprocessing

Log-scaled mel-spectrograms are more efficient for real-time implementation than CQT spectrograms, CWT scalograms, and MFCCs. Regarding model performance, some studies [35,38] show that using MFCCs as input to the model is better than the log-scaled mel-spectrograms, and some studies [22,23] show the opposite. Consequently, a simple comparison experiment was carried out with log-scaled mel-spectrograms and MFCCs. The result showed that using the log-scaled mel spectrograms as the input of the proposed model provides better performance. Thus, as mentioned above, the proposed model uses log-scaled mel-spectrograms as the input in the following experiment.

The parameters of data processing used were the same on the UrbanSound8K dataset and the HANS dataset. The audio signals consist of 16,000 samples per second. Specifically, log-scaled mel-spectrograms were extracted by the Librosa [39]. First, short-time Fourier transform spectrograms were generated with the hamming window size of 512, hop length of 256, and covering the audible frequency range (300–16,000 Hz). Second, the spectrograms were mapped to the 128 mel filters (bands). Then, the resulting spectrograms were converted to a logarithmic scale (decibels). Finally, the spectrograms were divided into multiple data by frame length. The data frame lengths of 60 (approximately 1 s), 90 (approximately 1.5 s), and 128 (approximately 2 s) represent Set A, Set B, and Set C, respectively. Many studies use different frame lengths, which could affect the classification accuracy. Thus, we used statistics from those studies and experiment with three frame lengths.

### 4.3. Data Augmentation

**Raw Data**. In order to avoid overfitting and to effectively utilize the limited data on UrbanSound8K, pitch-shifting [19] and time-stretching [19] deformation methods were used for each audio sample in order to generate new audio samples. These augmentations were applied using the MUDA [40] library. As shown in Table 2, the augmentation data of Set A, Set B, and Set C were increased to 13.2, 13.2, and 14.7 times, respectively, as compared with the corresponding original data.

**Figure 7:**
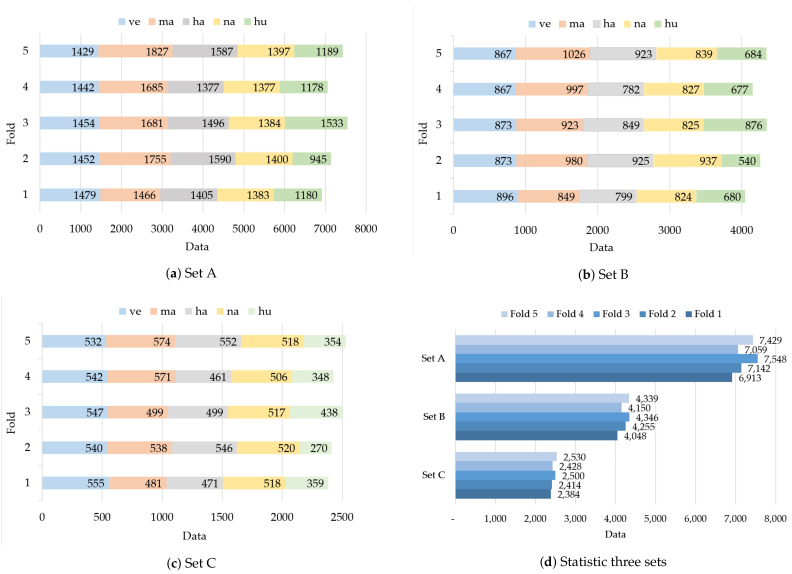
(**a**) The frame lengths of 60. (**b**) The frame lengths of 90. (**c**) The frame lengths of 128. (**d**) The statistic data of the three sets, shown distributed into five folds.

Because of the data imbalance problem in the HANS dataset, the pitch of each audio sample was shifted by a factor *r*. Next, the pitch-shifted sample was stretched for time by a random factor, to generate a new audio sample. The factor ranged from 0.9 to 1.2. However, the audio samples from the ESC50 dataset are fewer than those of from the UrbanSound8K dataset. Therefore, data augmentation was carried out with four sets of the relevant parameter in this research, as shown in Table 3. The differences between the four sets are the pitch-shifting factors and the amount of new audio samples. Table 4 presents the augmentation rules that apply to the four sets to balance the fold and class data. The augmentation was applied using the Librosa [39] library. The result after augmentation is illustrated in Figure 7.

**Spectrogram**. Mixup is a simple but effective method to generate training data [41], which we utilized. It uses mini-batch data which is selected from the whole of the original training data. Differently from traditional approaches, mixup generates new samples through the linear interpolation of two samples and their labels. New samples are determined by

(10) $$\begin{array}{c} \tilde{x}=\lambda x_{i}+\left(1-\lambda \right)x_{j},\\ \tilde{y}=\lambda y_{i}+\left(1-\lambda \right)y_{j}, \end{array}$$

where ($x_{i},y_{i}$) and ($x_{j},y_{j}$) are two samples randomly selected from training data. *x* represents raw input samples, *y* represents a one-hot label. The mix factor λ is a hyper-parameter α and λ∼ Beta(α,α). Note that mixup was only used for the training phase.

### 4.4. Training Settings

For the training stage, the Adam optimizer was used to train all models. The learning rate was initialized as 0.01 and reset as 0.01 when the learning rate was lower than 0.00001. The learning rate was automatically decreased by a factor of 0.5 when the validation loss stopped improving; after seven epochs for the UrbanSound8K dataset and five epochs for the HANS dataset. Every batch consisted of 32 samples randomly selected from the training set without repetition. The models were trained for 100 epochs and 50 epochs for UrbanSound8K and HANS, respectively.

For the testing stage, feature extraction and audio cropping patterns were the same as those used in the training stage. The k-fold cross-validation was used to evaluate the classification performance of the methods. For the UrbanSound8K dataset, k was set as 10 to produce a fair comparison with the results reported by Salmon et al. [38]. For the HANS dataset, k was set as 5 to validate mean accuracy.

All models were trained using the Keras [42] library with the TensorFlow backend on Nvidia GeForce RTX2080 with 16GB RAM. The inference time of all models was measured on the android mobile device Pixel 3a, and the models used TensorFlow Lite (TFLite). This mobile device possesses a Qualcomm Snapdragon 670 64-bit ARM-based octa-core system on a chip (SoC). The clock speed of this CPU varies between 1.7 and 2.0 GHz depending on the core being used. The internal memory of this smartphone is 4GB LPDDR4x RAM. It also possesses an Adreno 614 GPU. Note that TensorFlow for Mobile does not utilize this GPU.

## 5. Results

In this section, the performance of the proposed model and its classification results are shown. First, the performance of the proposed model on four conditions of the UrbanSound8k dataset are introduced. We provide comprehensive comparisons with other existing models [16,19,20,29] including the accuracy, the number of parameters, and total FLOPs. Afterward, the performances of the proposed model and three existing models [29,33,35] on the HANS dataset for noise classification is examined. We also provide various other comparisons with those models, including the accuracy, the number of parameters, total FLOPs, and inference time. Note that the android mobile device is used to measure the inference time.

### 5.1. Classification Results on Urbansound8k

**Figure 8:**
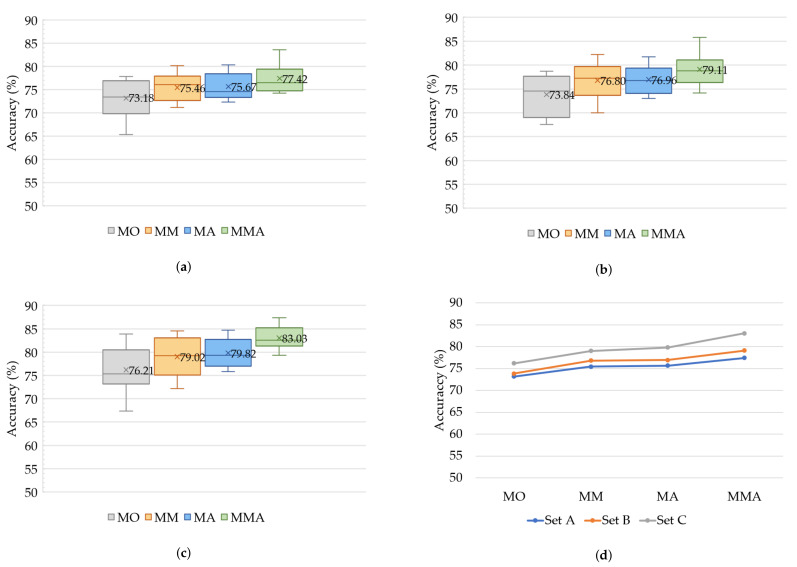
The classification results under four conditions of MO, MM, MA, and MMA and three sets Set A, Set B, and Set C. (**a**) The classification results of Set A. (**b**) The classification results of Set B. (**c**) The classification results of Set C. (**d**) The classification results of three sets.

Figure 8 shows the classification accuracy of the proposed model. The values in the figure are the average classification accuracy (%), which represent the rate of correct predictions on the total set of input data. MO represents the proposed model on the original data without data augmentation; MM represents the proposed model using the mixup technique on the original dataset; MA represents the proposed model on the augmentation dataset; MMA represents the proposed model using the mixup technique on the augmentation dataset. Figure 8a displays the classification results of Set A under four conditions. Comparing the accuracy of MO and other conditions, MM, MA, and MMA improvements are up to 2.28%, 2.49%, and 4.24%, respectively. Figure 8b displays the classification results of Set B under four conditions. Comparing the accuracy between MO and other conditions, MM, MA, and MMA improvements are up to 2.96%, 3.12%, and 5.27%, respectively. Figure 8c displays the classification results of Set C under four conditions. Comparing the accuracy between MO to other conditions—MM, MA, and MMA—the improvements are up to 2.81%, 3.61%, and 6.82%, respectively. Thus, data augmentation is shown to be an important technique for increasing the performance on a limited dataset. Moreover, the mixup technique is a powerful way to improve the classification accuracy in the training phase, resulting in better performance than for the model without mixup. Furthermore, when the two techniques are used simultaneously, the performance of the proposed model is at its best.

Figure 8d shows the average classification accuracy of three sets under four conditions. Set C produces the highest classification accuracy in the time division compared to other sets.With the increasing frame length of the spectrogram, the classification accuracy increases. Although greater sound length increases the number of frames, it provides more information about the environmental sound.

The mean classification accuracy of the proposed model compared with other existing models [16,19,20,29] is shown in Table 5. It can be observed that our method achieves a mean accuracy of 83.03% on UrbanSound8K. The mean classification accuracy of our method outperforms PizcakCNN [16] (baseline) and SBCNN [19] by 10.33% and 4.06%, respectively. In comparison with the ZhangCNN [29], the mean accuracy of the proposed model only increases 0.43%. However, the parameters and FLOPs of the proposed model are much lower than for ZhangCNN, indicating that the proposed model has significant advantages in computational efficiency over other models. Moreover, the mean classification accuracy of our method outperformed ResNet, Inception, and DenseNet by 9.77%, 7.79%, and 6.73%, respectively. The results show that our model, which uses the Inception-Dense block, is effective.

### 5.2. Classification Results on the Hearing Aids Noisy Sound (HANS) Dataset

**Figure 9:**
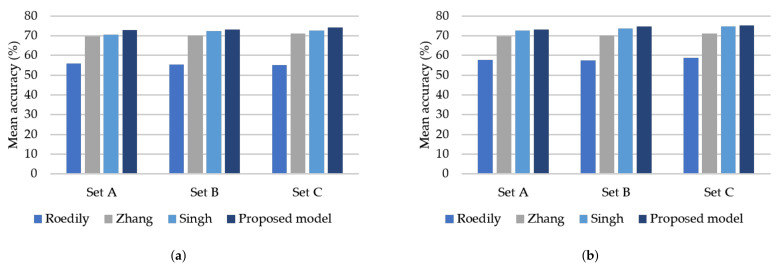
(**a**) The mean classification accuracy of models on the HANS dataset (**b**) The mean classification accuracy of models on the HANS dataset with the mixup technique. The frame length of Set A is 60, Set B is 90, and Set C is 128. The mean accuracy is the average score when performing 5-fold cross-validation.

The mean classification accuracy of the proposed model and three existing models [29,33,35] is presented in Figure 9 for noise classification. It can be observed that the proposed model is superior to other models due to its higher average classification accuracy. Figure 9a shows that Set C produces the highest classification accuracy compared to the other sets. The mean classification accuracy of Set A, Set B, and Set C can achieve the best precision by using the proposed model on the HANS dataset. The accuracies of Set A, Set B, and Set C are 72.73%,73.22%, and 74.03%, respectively. As a result, the proposed model is more effective than other models. Figure 9b shows that all models using the mixup technique significantly improved their accuracy. Compared with other models, the proposed model achieves the best accuracy in every Set. The improvements on Set A, Set B, and Set C are up to 0.47%, 1.62%, and 1.24%, respectively. Thus, we can state that the mixup technique is helpful on the HANS dataset.

Table 6 presents the performance of different architectures on the HANS dataset. It is apparent that the proposed model has the best accuracy and the fewest parameters among all models. The FLOPs of the proposed model are the second least of all models. Although the Roedily model has fewer FLOPs than those of our model, the accuracy of our model is much higher. Therefore, the proposed model can use fewer parameters and FLOPs while achieving better accuracy and inference time.

## 6. Conclusions

In this article, we present a specially designed network for accurately recognizing urban and noise sounds. The proposed model aims to take full advantage of the low-level information in log-scaled mel-spectrograms to make its classification decisions. This architecture is shown to be competitive with other deep learning architectures evaluated on the UrbanSound8K dataset and our HANS dataset. Moreover, the input spectrograms are generated using three different frame lengths. We found that the classification accuracy of the proposed model gradually increased with an increased frame length of the input spectrogram. Thus, we can deduce that more information can be obtained due to the longer audio frame length.

Additionally, the mixup technique is used to increase data diversity in the training phase. The experimental results showed that the mixup technique could enhance classification accuracy. The proposed model performed better than other models on two datasets, and the inference time of the proposed model is short. Despite the proposed model’s performance being competitive, there are limitations to improving the accuracy of the proposed model on the HANS dataset. Thus, we plan to use different input-transform methods and other data-augmentation methods to further improve the proposed model in our future work. The proposed noise classifier will be used as part of a noise reduction app for hearing-improvement purposes.

## Figures and Tables

**Table 1:** The configuration of the proposed model. Out Shape represents the dimension in (frame,melfeatures,channel). The model only has 97 k parameters.

Layer	Filter Size/Stride (Number of Filters)	Out Shape	Params
Input	–	(128, 128, 1)	
Conv1	3×3/1 (16)	(128, 128, 16)	228
Conv2	3×3/1 (16)	(128, 128, 16)	2,384
MaxPool1	3×3/1 (None)	(42, 42, 16)	
Inception (a)	[1×1/1(16)conv]*1, [1×1/1(16)conv, 3×3/1(32)conv]*1, [1×1/1(16)conv, 3×3/1(32)conv, 3×3/1(32)]*1, [3×3/1(None)avergepool,1×1/1(16)conv]	(42, 42, 112)	20,128
Inception (b)	[1×1/1(32)conv]*1, [1×1/1(32)conv, 3×3/1(32)conv]*1, [1×1/1(32)conv, 3×3/1(32)conv, 3x3/1(32)conv]*1, [3×3/1(None)avergepool, 1×1/1(32)conv]	(42, 42, 240)	21,152
Inception (c)	[1×1/1(32)conv]*1, [1×1/1(32)conv, 3×3/1(32)conv]*1, [1×1/1(32)conv, 3×1/1(32)conv, 3×3/1(32)conv]*1, [3×3/1(None)avergepool,1×1/1(32)conv]	(42, 42, 368)	39,584
Conv3	1×1/1(32)	(42, 42, 32)	13,408
AveragePool	2×2/2×2 (None)	(42, 10, 32)	
GlobalAvergePool1	–	(32)	
Dense	number of classes	number of classes	330
	Total Params		97,214

**Table 2:** The UrbanSound8K dataset is divided into three sets by the original data and the augmentation data.

Fold	UrbanSound8K			UrbanSound8k (aug)		
	SetA	SetB	SetC	SetA	SetB	SetC
1	5238	2619	873	69,593	34,792	12,841
2	5328	2664	888	70,740	35,372	12,964
3	5550	2775	925	73,766	36,885	13,528
4	5940	2970	990	78,853	39,429	14,390
5	5616	2808	936	74,556	37,280	13,555
6	4938	2469	823	65,599	32,800	11,966
7	5028	2514	838	66,804	33,405	12,250
8	4836	2418	806	64,211	32,114	11,964
9	4896	2448	816	65,035	32,519	12,118
10	5022	2511	837	66,698	33,350	12,376

**Table 3:** The description of four groups.

Group	Sample	*r*
1.5	Even	2
2	Each	2
5	Each	−2.5, −2, −1, 1, 2
6	Each	−2.5, −2, −1, 1, 2, 2.5

**Table 4:** The HANS dataset augmentation rule.

Major Categorie	Class	Fold 1	Fold 2	Fold 3	Fold 4	Fold 5
Vehicle engine (ve)	Helicopter	6	6	6	6	6
	Airplane	6	6	6	6	6
	Train	6	6	6	6	6
Machine tools (ma)	Drilling	None	None	None	2	1.5
	Jackhammer	2	None	None	1.5	None
	Chainsaw	6	6	6	6	6
Household appliance (ha)	Air condition	None	1.5	None	None	1.5
	Vacuum cleaner	6	6	6	6	6
	Washing machine	6	6	6	6	6
Natural (na)	Thunderstorm	5	5	5	5	5
	Sea waves	5	5	5	5	5
	Rain	5	5	5	5	5
	Wind	5	5	5	5	5
Human speech (hu)	Children playing	None	None	None	None	None
	Street music	1.5	None	None	2	2

**Table 5:** Comparison of classification accuracy with other models on UrbanSound8K dataset. The bold number is our result.

Approach	Mean Acc	Parameters	FLOPs
PiczakCNN [16]	73.09	109,134,090	515,806,780
ResNet [20]	73.26	23,608,202	5,044,643,516
Inception [20]	75.24	21,823,274	3,196,935,548
DenseNet [20]	76.30	18,341,194	5,367,216,252
SBCNN [19]	79.00	874,746	170,694,732
ZhangCNN [29]	82.60	1,186,322	882,779,336
Proposed model	**83.03**	**97,214**	**394,483,170**

**Table 6:** Comparison of the four models on HANS dataset. The bold is our result.

Approach	Param	Set	Acc (%)	FLOPs	Inference Time (s)	
					Model	FLM
Roediy [35]	116,869	A	57.70	6,184,606	0.012	0.033
		B	57.48	9,234,590	0.023	0.045
		C	58.72	13,191,710	0.036	0.057
Zhang [29]	1,183,081	A	71.29	377,229,476	0.064	0.084
		B	72.56	582,501,796	0.093	0.112
		C	71.01	860,721,316	0.130	0.146
Singh [33]	4,694,473	A	72.53	3,072,945,444	0.264	0.283
		B	73.78	4,637,731,620	0.391	0.408
		C	74.75	6,676,496,676	0.549	0.565
Proposed model	**97,049**	A	**73.20**	187,247,748	0.032	0.061
		B	**74.84**	280,86,4388	0.049	0.067
		C	**75.27**	394,482,820	0.067	0.084

FLM: First Load tflite Model on mobile device.

## Data Availability

The data presented in this study are available in this published article.
